# Supplementary material for: Temporal and context-dependent requirements for the transcription factor Foxp3 expression in regulatory T cells
Source: Nat Immunol. 2025 Oct 8;26(11):2059–73. doi: 10.1038/s41590-025-02295-4 (PMC12571910; doi:10.1038/s41590-025-02295-4)
Supplement: Supplementary file 1 — Reporting Summary [file 41590_2025_2295_MOESM1_ESM.pdf]

nature portfolio

Corresponding author(s): Alexander Rudensky, Wei Hu, Yuri Prytikin

Last updated by author(s): Aug 23, 2025

Reporting Summary

Nature Portfolio wishes to improve the reproducibility of the work that we publish. This form provides structure for consistency and transparency in reporting. For further information on Nature Portfolio policies, see our Editorial Policies and the Editorial Policy Checklist.

Statistics

For all statistical analyses, confirm that the following items are present in the figure legend, table legend, main text, or Methods section.

n/a	Confirmed	
☐	☒	The exact sample size (*n*) for each experimental group/condition, given as a discrete number and unit of measurement
☐	☒	A statement on whether measurements were taken from distinct samples or whether the same sample was measured repeatedly
☐	☒	The statistical test(s) used AND whether they are one- or two-sided<br>*Only common tests should be described solely by name; describe more complex techniques in the Methods section.*
☐	☒	A description of all covariates tested
☐	☒	A description of any assumptions or corrections, such as tests of normality and adjustment for multiple comparisons
☐	☒	A full description of the statistical parameters including central tendency (e.g. means) or other basic estimates (e.g. regression coefficient) AND variation (e.g. standard deviation) or associated estimates of uncertainty (e.g. confidence intervals)
☐	☒	For null hypothesis testing, the test statistic (e.g. *F*, *t*, *r*) with confidence intervals, effect sizes, degrees of freedom and *P* value noted<br>*Give P values as exact values whenever suitable.*
☒	☐	For Bayesian analysis, information on the choice of priors and Markov chain Monte Carlo settings
☒	☐	For hierarchical and complex designs, identification of the appropriate level for tests and full reporting of outcomes
☐	☒	Estimates of effect sizes (e.g. Cohen's *d*, Pearson's *r*), indicating how they were calculated

Our web collection on statistics for biologists contains articles on many of the points above.

Software and code

Policy information about availability of computer code

Data collection	Flow cytometry data were collected on an Aurora cytometer (Cytek) using SpectroFlo v3.1.2 (Cytek).<br>ELISA data were recorded with Synergy HTX plate reader with Gen5 v3.02.2(BioTek).<br>Bulk RNA-seq libraries were sequenced on NovaSeq 6000 (Illumina).<br>scRNA-seq and scATAC-seq libraries were sequenced on an NovaSeq 6000 System (Illumina).
Data analysis	Flow cytometry data were analyzed using FlowJo v10.6.1 (BD).<br>ELISA data were calculated with Gen5 3.02.2 (BioTek).<br>Statistical analyses of biological experiments were performed using Prism v10.0.<br>For RNA-seq experiment, STAR aligner and Genome Analysis Toolkit were used for alignment, R was used for measuring the raw count of reads per gene, and the DESeq2 R package was used to perform differential gene expression analysis.<br>For scRNA-seq, fastq files were processed using Cell Ranger (10x Genomics).<br>DNA sequence analysis was performed using SnapGene v8.0.1.<br>The code used for computational analysis is available at https://github.com/pritykinlab/scTir1_Analysis . The lead contacts can provide and any additional information required to reanalyze the data reported in this paper upon request.

For manuscripts utilizing custom algorithms or software that are central to the research but not yet described in published literature, software must be made available to editors and reviewers. We strongly encourage code deposition in a community repository (e.g. GitHub). See the Nature Portfolio guidelines for submitting code & software for further information.

Data

Policy information about availability of data

All manuscripts must include a data availability statement. This statement should provide the following information, where applicable:

- Accession codes, unique identifiers, or web links for publicly available datasets
- A description of any restrictions on data availability
- For clinical datasets or third party data, please ensure that the statement adheres to our policy

RNA-seq, and ATAC-seq data generated in this study have been deposited in GEO and are publicly available. Accession numbers are listed below.
Bulk RNA-seq: GSE297451
Bulk ATAC-seq: GSE297597
Tumor scRNA-seq: GSE297598
Tissue Treg and adoptive transfer scRNA-seq: GSE297622

Research involving human participants, their data, or biological material

Policy information about studies with human participants or human data. See also policy information about sex, gender (identity/presentation), and sexual orientation and race, ethnicity and racism.

Reporting on sex and gender	*Use the terms sex (biological attribute) and gender (shaped by social and cultural circumstances) carefully in order to avoid confusing both terms. Indicate if findings apply to only one sex or gender; describe whether sex and gender were considered in study design; whether sex and/or gender was determined based on self-reporting or assigned and methods used. Provide in the source data disaggregated sex and gender data, where this information has been collected, and if consent has been obtained for sharing of individual-level data; provide overall numbers in this Reporting Summary. Please state if this information has not been collected. Report sex- and gender-based analyses where performed, justify reasons for lack of sex- and gender-based analysis.*
Reporting on race, ethnicity, or other socially relevant groupings	*Please specify the socially constructed or socially relevant categorization variable(s) used in your manuscript and explain why they were used. Please note that such variables should not be used as proxies for other socially constructed/relevant variables (for example, race or ethnicity should not be used as a proxy for socioeconomic status). Provide clear definitions of the relevant terms used, how they were provided (by the participants/respondents, the researchers, or third parties), and the method(s) used to classify people into the different categories (e.g. self-report, census or administrative data, social media data, etc.) Please provide details about how you controlled for confounding variables in your analyses.*
Population characteristics	*Describe the covariate-relevant population characteristics of the human research participants (e.g. age, genotypic information, past and current diagnosis and treatment categories). If you filled out the behavioural & social sciences study design questions and have nothing to add here, write "See above."*
Recruitment	*Describe how participants were recruited. Outline any potential self-selection bias or other biases that may be present and how these are likely to impact results.*
Ethics oversight	*Identify the organization(s) that approved the study protocol.*

Note that full information on the approval of the study protocol must also be provided in the manuscript.

Field-specific reporting

Please select the one below that is the best fit for your research. If you are not sure, read the appropriate sections before making your selection.

☒ Life sciences ☐ Behavioural & social sciences ☐ Ecological, evolutionary & environmental sciences

For a reference copy of the document with all sections, see nature.com/documents/nr-reporting-summary-flat.pdf

Life sciences study design

All studies must disclose on these points even when the disclosure is negative.

Sample size	No sample size calculation was performed. Whenever possible at least 2 independent biological replicates were analyzed. In vivo experiments were performed at least twice to ensure reproducibility.
Data exclusions	In the bulk RNA-seq experiment in Figure 2, three biological replicates of each cell type were generated but Foxp3-AID R26-TIR1 resting Treg ended up being represented by only two because the third replicate seemed to be an outlier based on principal component analysis.
Replication	All in vivo experiments were repeated at least twice and all attempts at replication were successful.
Randomization	Mice were grouped according to genotype and all experiments were performed with sex-matched littermates.

Blinding

For pathology scoring, the pathologist was blinded from the genotype and treatment of the mice.

Reporting for specific materials, systems and methods

We require information from authors about some types of materials, experimental systems and methods used in many studies. Here, indicate whether each material, system or method listed is relevant to your study. If you are not sure if a list item applies to your research, read the appropriate section before selecting a response.

Materials & experimental systems

n/a	Involved in the study
☐	☒ Antibodies
☐	☒ Eukaryotic cell lines
☐	☐ Palaeontology and archaeology
☐	☒ Animals and other organisms
☐	☐ Clinical data
☐	☐ Dual use research of concern
☐	☐ Plants

Methods

n/a	Involved in the study
☐	☐ ChIP-seq
☐	☒ Flow cytometry
☐	☐ MRI-based neuroimaging

Antibodies

Antibodies used

The following antibodies and reagents were used in this study for flow cytometry, with clones, venders, catalog numbers and dilutions as indicated: anti-Siglec-F (E50-2440, BD, 562681, 1:400), anti-I-A/I-E (M5/114.15.2, Biosciences, 566086, 1:1,200), anti-NK1.1 (PK136, Thermo Fisher, 47-5941-82, 1:400), anti-CD45 (30-F11, BioLegend, 103136, 1:600), anti-CD11b (M1/70, BioLegend, 101257, 1:800), anti-CD11b (M1/70, BD Biosciences, 363-0112-82, 1:400), anti-CD3ε (17A2, BioLegend, 100237, 1:500), anti-γδTCR (GL3, BD Biosciences, 750410, 1:300), anti-Cd278 (C398.4A, BD Biosciences, 567918, 200), anti-TCR beta (H57-597, BD Biosciences, 748405, 1:300), Anti-TCR beta (H57-597, Thermo Fisher, 47-5961-82, 1:300), anti-TCR beta (H57-597, BioLegend, 109227, 1:200), anti-TCR beta (H57-597, Thermo Fisher, 12-5961-83, 1:400), anti-CD153 (RM153, BD Bioscience, 741575, 1:400), anti-CD24 (M1/69, Thermo Fisher, 46-0242-82, 1:800), anti-CD304 (3E12, BioLegend, 145209, 1:300), anti-CD3 (IM7, BioLegend, 103049, 1:400), anti-CD44 (IM7, BD Biosciences, 563971, 1:400), anti-CD44 (IM7, BioLegend, 103026, 1:100), anti-ZsGreen (polyclonal, Frontier Institute co.,ltd, MSFR106470, 1:800), anti-KLRG1 (2F1, Thermo Fisher, 35-5893-82), anti-CD39 (24DMS1, Thermo Fisher, 25-0391-82, 1:400), anti-TCF1 (C63D9, Cell signaling, 6709, 1:200), anti-IL-10 (JES5-16E3, BioLegend, 505021, 1:200), anti-CD4 (RM4-5, BD Biosciences, 414-0042-82, 1:400), anti-CD4 (RM4-5, BioLegend, 100536, 1:400), anti-CD4 (RM4-5, Thermo Fisher, 47-0042-82, 1:400), anti-CD4 (RM4-5, BioLegend, 100548, 1:400), anti-TNFa (MP6-XT22, BioLegend, 506329, 1:400), anti-IFNg (XMG1.2, BioLegend, 505836, 1:200), anti-IL-22 (1H8PWSR, Thermo Fisher, 46-7221-80, 1:400), anti-IL13 (eBio13A, Thermo Fisher, 12-7133-82, 1:400), anti-IL-4 (11B11, Thermo Fisher, 17-7041-82, 1:300), anti CD11c (N418, Thermo Fisher, 48-0114-82, 1:200), anti-Ly6c (HK1.4, BioLegend, 128037, 1:1,200), anti-Ly6C (HK1.4, BioLegend, 128041, 1:1000), anti-CD122 (TM-β1, BD Bioscience, 564763, 1:200), anti-GARP (YGIC86, Thermo Fisher, 25-9891, 1:200), anti-CD86 (GL1, Thermo Fisher, 12-0862-85, 1:400), anti-Ly6G (1A8, BioLegend, 127618, 1:500), anti-CD64 (X54-5/7.1, BioLegend, 139306, 1:200), anti-CD127 (A7R34, Tonbo Bioscience, 20-1271-U100, 1:200), anti-CD122 (5H4, Thermo Fisher, 13-1221-82, 1:200), anti-Guinea Pig (polyclonal, Thermo Fisher, SA5-10094, 1:1,000), anti-FR4 (12A5, BD Biosciences, 744121, 1:200), anti-FR4 (12A5, BD Biosciences, 560318, 1:200), anti-OX40 (OX-86, Thermo Fisher, 46-1341-82, 1:300), anti-CD120b (TR75-89, BD Bioscience, 564088, 1:200, anti-CD103 (M290, BD Biosciences, 566118, 1:300), anti-Ly-6C (HK1.4, BioLegend, 128037, 1:1,000), anti-CD90.2 (30-H12, BioLegend, 105320, 1:800), anti-CD90.2 (53-2.1, BD Biosciences, 564365, 1:1,500), anti-Foxp3 (FJK-16s, Thermo Fisher, 48-5773-82, 1:200), anti-Foxp3 (FJK-16s, Thermo Fisher, 17-5773-82, 1:200), anti-CD19 (6D5, BioLegend, 115510, 1:600), anti-F4|80 (BM8, BioLegend, 123133, 1:200), anti-CD4 (RM4-5, EBioscience, 564667, 1:400), anti-CD4 (RM4-5, BioLegend, 100553, 1:400), anti-CD8α (53-6.7, BioLegend, 100780, 1:600), anti-CD8α (53-6.7, BioLegend, 564297, 1:400), anti-CD8α (53-6.7, BioLegend, 100752, 1:500), anti-GITR (DTA-1, Thermo Fisher, 48-5874-82, 1:500), anti-CD73 (eBioTY/11.8, Thermo Fisher, 46-0731-82, 1:400), anti-CD73 (TY/11.8, BioLegend, 127208, 1:400), anti-CD62L (MEL-14, BioLegend, 104441, 1:100), anti-CD62L (MEL-14, BD Biosciences, 565213, 1:600), anti-CD62L (MEL-14, BD Biosciences, 741230, 1:800), anti-CD62L (MEL-14, BioLegend, 104441, 1:400), anti-CD62L (MEL-14, BioLegend, 104438, 1:1,600), anti-CTLA4 (UC10-4B9, BioLegend, 106323, 1:200), anti-CTLA4 (UC10-4B9, Thermo Fisher, 12-1522-82, 1:400), anti-Helios (22F6, BioLegend, 137216, 1:400), anti-Helios (22F6, BioLegend, 137236, 1:400), anti-Eos (ESB7C2, Thermo Fisher, 12-5758-82, 1:400), anti-Ki-67 (SolA15, Thermo Fisher, 61-5698, 1:2,000), anti Ki67 (B56, BD Biosciences, 563757, 1:1,000), anti-Ki67 (SolA15, Fisher Scientific, 15-5698-82, 1:8,000), anti-CD25 (PC61, BD Biosciences, 564022, 1:300; Thermo Fisher, 17-0251-82, 1:400), anti-PD-1 (29F.1A12, BioLegend, 135225, 1:400), anti-CD45 (30-F11, BioLegend, 103157, 1:1,000), anti-IL-2 (JES6-5H4, BioLegend, 503818, 1:400), streptavidin (Thermo Fisher, 46-4317-82, 1:1,000), Picolyl-Azide (Jena Bioscience, CLK-1288-5), CTV (Thermo Fisher, C34557), Zombie NIR dye (BioLegend, 423105, 1:1,000), Sytox Blue (Thermo Fisher, S34857), anti-mouse CD16/32 (2.4G2, Tonbo, 70-0161-M001, 1:500).

The following antibodies were used for ELISA capturing in this study: Purified anti-mouse IL-13 (14-7133-68, Invitrogen, 88-7137-88), Purified anti-mouse IL-4 (14-7041-68A, Invitrogen, 88-7044-88), Purified anti-mouse IL-2 (eBioscience, 14-7022-68). Purified anti-mouse IgE (R35-72, BD Pharmingen, 553413), goat anti-mouse IgG1 (2794408, Southern Biotech, 1070-01), Goat Anti-Mouse IgG3 (2794567, Southern Biotech, 1100-01), Goat anti-mouse IgG2a (2794475, Southern Biotech, 1080-01), goat anti-mouse IgG2b (2794517, Southern Biotech, 1090-01), Goat Anti-Mouse IgG2c (2794464, Southern Biotech, 1079-01), goat anti-mouse IgA (2314669, Southern Biotech, 1040-01), goat anti-mouse IgM (2794197, Southern Biotech, 1020-01).

The following antibodies were used for ELISA detection in this study: biotin anti-mouse IL-13 (13-7135-68A, Invitrogen, 88-7137-88), anti-mouse IL-4 (13-7042-68C, Invitrogen, 88-7044-88), anti-mouse IL-2 (eBioscience, 33-7021-68), Goat Anti-Mouse Ig (2728714, Southern Biotech, 1010-05), biotin rat anti-mouse IgE (R35-118, BD Pharmingen, 553419).

The following reagents were used to construct standard curves for ELISA in this study: mouse IL-4 lyophilized standard (39-8041-60, Invitrogen, 88-7044-88), mouse IL-13 lyophilized standard (39-7137/2EB-60, Invitrogen, 88-7137-88), mouse IL-2 (Thermo Fisher 212-12-5UG), purified Mouse IgG1, kappa, Isotype Control (15H6, Southern Biotech, 0102-01), purified mouse IgG2a, kappa, Isotype Control (UPC-10, Sigma, M5409), IgG2b Isotype Control from murine myeloma (MOPC-141, Sigma, M5534), Mouse IgG2c (6.3, AB_2794064, Southern Biotech, 0122-01), Purified Mouse IgG3, kappa, Isotype Control (A112-3, BD Pharmingen, 553486), Purified Mouse IgA, kappa, Isotype Control (M18-254, BD Pharmingen, 553476), IgM Isotype Control from murine myeloma (MOPC 104E, Sigma, M5909), Purified Mouse IgE, kappa, Isotype Control (C38-2, BD Pharmingen, 557079).

All antibodies are routinely QC'ed by the manufacturer for their specificity. The optimal dilution factor is determined in house by titration.

Validation	All above antibodies are well validated commercial clones or preps routinely QC'ed by the manufacturer.

Eukaryotic cell lines

Policy information about cell lines and Sex and Gender in Research

Cell line source(s)	293T cells were originally purchased from ATCC (CRL-3216). B16-OVA cells were gifted by James Allison.
Authentication	Cells were not authenticated. However, the 293 T cells maintained their ability to produce high titer retrovirus throughout the study, and the B16-OVA cells maintained their ability to form a black tumor subcutaneously.
Mycoplasma contamination	Cells were routinely tested for mycoplasma contamination and remained negative throughout the study.
Commonly misidentified lines (See ICLAC register)	NA

Palaeontology and Archaeology

Specimen provenance	*Provide provenance information for specimens and describe permits that were obtained for the work (including the name of the issuing authority, the date of issue, and any identifying information). Permits should encompass collection and, where applicable, export.*
Specimen deposition	*Indicate where the specimens have been deposited to permit free access by other researchers.*
Dating methods	*If new dates are provided, describe how they were obtained (e.g. collection, storage, sample pretreatment and measurement), where they were obtained (i.e. lab name), the calibration program and the protocol for quality assurance OR state that no new dates are provided.*

☐ Tick this box to confirm that the raw and calibrated dates are available in the paper or in Supplementary Information.

Ethics oversight	*Identify the organization(s) that approved or provided guidance on the study protocol, OR state that no ethical approval or guidance was required and explain why not.*

Note that full information on the approval of the study protocol must also be provided in the manuscript.

Animals and other research organisms

Policy information about studies involving animals; ARRIVE guidelines recommended for reporting animal research, and Sex and Gender in Research

Laboratory animals	Foxp3-AID R26-WT, Foxp3-AID R26-TIR1, Foxp3-fl, Cd4-creERT2, Foxp3-DTR, TCRbd double knockout, C57BL/6, and Foxp3 knockout mice were used at 1 day or 6-10 weeks of age as described in the manuscript. All experiments were performed using sex-matched littermate controls whenever possible.
Wild animals	*Provide details on animals observed in or captured in the field; report species and age where possible. Describe how animals were caught and transported and what happened to captive animals after the study (if killed, explain why and describe method; if released, say where and when) OR state that the study did not involve wild animals.*
Reporting on sex	Both male and female mice were used in this study. Because Foxp3 is an X chromosome linked gene, Foxp3-AID/WT heterozygous female mice were used to eliminate cell-extrinsic effects upon Foxp3 degradation (Figure 2, Figure 3, Figure 4a-d, Figure 5a-f). Foxp3AID/y hemizygous males were used in other experiments to assess the immunological consequence of Foxp3 degradation in vivo (Figure 1, Figure 5g-o, Figure 6, Figure 7).
Field-collected samples	NA
Ethics oversight	All animal experiments in this study were approved by the Sloan Kettering Institute Institutional Animal Care and Use Committee under protocol 08-10-023 or Yale University Institutional Animal Care and Use Committee under protocol 2023-20503.

Note that full information on the approval of the study protocol must also be provided in the manuscript.

Clinical data

Policy information about clinical studies

All manuscripts should comply with the ICMJE guidelines for publication of clinical research and a completed CONSORT checklist must be included with all submissions.

Clinical trial registration	*Provide the trial registration number from ClinicalTrials.gov or an equivalent agency.*
Study protocol	*Note where the full trial protocol can be accessed OR if not available, explain why.*
Data collection	*Describe the settings and locales of data collection, noting the time periods of recruitment and data collection.*
Outcomes	*Describe how you pre-defined primary and secondary outcome measures and how you assessed these measures.*

Dual use research of concern

Policy information about dual use research of concern

Hazards

Could the accidental, deliberate or reckless misuse of agents or technologies generated in the work, or the application of information presented in the manuscript, pose a threat to:

No	Yes	
☒	☐	Public health
☒	☐	National security
☒	☐	Crops and/or livestock
☒	☐	Ecosystems
☒	☐	Any other significant area

Experiments of concern

Does the work involve any of these experiments of concern:

No	Yes	
☒	☐	Demonstrate how to render a vaccine ineffective
☒	☐	Confer resistance to therapeutically useful antibiotics or antiviral agents
☒	☐	Enhance the virulence of a pathogen or render a nonpathogen virulent
☒	☐	Increase transmissibility of a pathogen
☒	☐	Alter the host range of a pathogen
☒	☐	Enable evasion of diagnostic/detection modalities
☒	☐	Enable the weaponization of a biological agent or toxin
☒	☐	Any other potentially harmful combination of experiments and agents

Plants

Seed stocks	*Report on the source of all seed stocks or other plant material used. If applicable, state the seed stock centre and catalogue number. If plant specimens were collected from the field, describe the collection location, date and sampling procedures.*
Novel plant genotypes	*Describe the methods by which all novel plant genotypes were produced. This includes those generated by transgenic approaches, gene editing, chemical/radiation-based mutagenesis and hybridization. For transgenic lines, describe the transformation method, the number of independent lines analyzed and the generation upon which experiments were performed. For gene-edited lines, describe the editor used, the endogenous sequence targeted for editing, the targeting guide RNA sequence (if applicable) and how the editor was applied.*
Authentication	*Describe any authentication procedures for each seed stock used or novel genotype generated. Describe any experiments used to assess the effect of a mutation and, where applicable, how potential secondary effects (e.g. second site T-DNA insertions, mosiacism, off-target gene editing) were examined.*

ChIP-seq

Data deposition

☐ Confirm that both raw and final processed data have been deposited in a public database such as GEO.

☐ Confirm that you have deposited or provided access to graph files (e.g. BED files) for the called peaks.

Data access links<br>*May remain private before publication.*	*For "Initial submission" or "Revised version" documents, provide reviewer access links. For your "Final submission" document, provide a link to the deposited data.*
Files in database submission	*Provide a list of all files available in the database submission.*
Genome browser session<br>(e.g. UCSC)	*Provide a link to an anonymized genome browser session for "Initial submission" and "Revised version" documents only, to enable peer review. Write "no longer applicable" for "Final submission" documents.*

Methodology

Replicates	*Describe the experimental replicates, specifying number, type and replicate agreement.*
Sequencing depth	*Describe the sequencing depth for each experiment, providing the total number of reads, uniquely mapped reads, length of reads and whether they were paired- or single-end.*
Antibodies	*Describe the antibodies used for the ChIP-seq experiments; as applicable, provide supplier name, catalog number, clone name, and lot number.*
Peak calling parameters	*Specify the command line program and parameters used for read mapping and peak calling, including the ChIP, control and index files used.*
Data quality	*Describe the methods used to ensure data quality in full detail, including how many peaks are at FDR 5% and above 5-fold enrichment.*
Software	*Describe the software used to collect and analyze the ChIP-seq data. For custom code that has been deposited into a community repository, provide accession details.*

Flow Cytometry

Plots

Confirm that:

☐ The axis labels state the marker and fluorochrome used (e.g. CD4-FITC).

☒ The axis scales are clearly visible. Include numbers along axes only for bottom left plot of group (a 'group' is an analysis of identical markers).

☒ All plots are contour plots with outliers or pseudocolor plots.

☒ A numerical value for number of cells or percentage (with statistics) is provided.

Methodology

Sample preparation

Animals were euthanized and perfused with 20 ml PBS. Cells were isolated form the lymphoid organs by meshing with the end of a syringe plunger through a 100 mm cell strainer (Corning, 07-201-432). Lungs and tumors were digested in RPMI 1640 with 2% FBS,10 mM HEPES buffer, 100 U/mL penicillin-streptomycin, 2 mM L-glutamate, 0.2 U/mL collagenase A (Sigma, 11088793001) and 1U/mL DNase I (Sigma-Aldrich, 10104159001) for 45 min at 37°C with vigorous shaking at 250 r.p.m. 6.35mm ceramic beads (MP Biomedicals, 116540034) were included to help with tissue dissociation. The digested lungs and tumors were filtered through 70 mm separation filters (Miltenyi Biotec, 130-095-823), washed and centrifuged in PBS-adjusted 40% Percoll (Sigma Aldrich, 17-0891-01) to enrich for lymphocytes. Erythrocytes from spleen, lung and tumor were lysed by using of ACK lysis buffer (150 mM NH_4Cl (Sigma-Aldrich, A9434), 10 mM $KHCO_3$ (Sigma-Aldrich, P7682) and 0.1 mM Na_2EDTA at pH 7.4).

For flow cytometry staining cells were stained with Zombie NIR dye in PBS for 10 min at 4°C to identify the dead cells followed by staining with anti-mouse CD16/32 in Staining Buffer (PBS with 0.2% BSA, 10 mM HEPES buffer and 2 mM EDTA) for 10 min at 4°C to block the Fc receptors. Next, cells were stained with fluorescently conjugated antibodies detecting cell surface antigens for 30 minutes at 4°C. To access the intracellular antigens, cells were fixed and permeabilized with eBioscience transcription factor staining buffer set (00-5523-00) according to the manufacturer's instructions.

To measure cytokine production following ex-vivo stimulation, single cell suspension was incubated with 5% CO_2 at 37°C for 4 hours in the cell culture media (RPMI 1640 media supplied with 10% FBS, 100 U/mL penicillin–streptomycin, 2mM L-Glutamine, 10 mM HEPES buffer, 50 µM β-mercaptoethanol) supplied with 50ng/ml phorbol-12-myristate-13-acetate (Sigma-Aldrich, P8139), 500ng/ml ionomycin (Sigma-Aldrich, I0634), 2µM monensin (Sigma-Aldrich, M5273), 1µg/ml brefeldin A (Sigma-Aldrich, B6542). Cells were stained for flow cytometry as described above except for the fixation/permeabilization step and cytokine staining in which case BD Cytofix/Cytoperm Kit (BD Biosciences, 554715) was used according to the manufacturer's instructions.

Instrument	Flow cytometry data was collected on a 5-laser Aurora cytometer (Cytek)
Software	Flow cytometry data was recorded using SpectroFlo software v3.1.2 and analyzed using FlowJo v10.10.0.
Cell population abundance	Post sort purity was routinely examined by flow cytometry. scRNA-seq and scATAC-seq, as well as functional assay samples were sorted to a purity of at least 95%. Bulk RNA-seq samples were double sorted to a purity of at least 99%.
Gating strategy	Gating strategy of Foxp3-AID Treg cells:<br>1. Lymphocyte gating based on FSC-A and SSC-A.<br>2. Singlet gating based on FSC-W and FSC-H, followed by SSC-W and SSC-H.<br>3. Gate on CD4 T cells: CD4+TCRb+.<br>4. Gate on Treg cells: CD4+TCRb+ZsGreen+ from R26-WT mice; CD4+TCRb+ZsGreen+mCherry+ from R26-TIR1(F74G) mice.<br>5. In some experiments, resting (CD62hiCD44lo) and activated (CD62LloCD44hi) Treg cells were sorted separately.<br><br>Gating strategy for naive CD4 T cells from Foxp3-DTR mice:<br>1. Lymphocyte gating based on FSC-A and SSC-A.<br>2. Singlet gating based on FSC-W and FSC-H, followed by SSC-W and SSC-H.<br>3. Gate on CD4 T cells: CD4+TCRb+.<br>4. Gate on naive CD4 T cells: CD4+TCRb+GFP-CD62hiCD44lo.<br><br>Gating strategy for Treg wannabe cells with and without Foxp3-restoration.<br>1. Lymphocyte gating based on FSC-A and SSC-A.<br>2. Singlet gating based on FSC-W and FSC-H, followed by SSC-W and SSC-H.<br>3. Gate on CD4 T cells: CD4+CD3+TCRb+.<br>4. Treg wannabe cells from Foxp3-LSL Cd4-WT mice were gate as Thy1.1+GFP-; Foxp3 restored Treg wannabe cells from Foxp3-LSL Cd4-creERT2 mice were gated as CD4+TCRb+Thy1.1+/-GFP+.<br>5. Resting (CD62hiCD44lo) and activated (CD62LloCD44hi) Treg wannabe cells (with or without Foxp3 restoration) were sorted separately.

☒ Tick this box to confirm that a figure exemplifying the gating strategy is provided in the Supplementary Information.

Magnetic resonance imaging

Experimental design

Design type	*Indicate task or resting state; event-related or block design.*
Design specifications	*Specify the number of blocks, trials or experimental units per session and/or subject, and specify the length of each trial or block (if trials are blocked) and interval between trials.*
Behavioral performance measures	*State number and/or type of variables recorded (e.g. correct button press, response time) and what statistics were used to establish that the subjects were performing the task as expected (e.g. mean, range, and/or standard deviation across subjects).*

Acquisition

Imaging type(s)	*Specify: functional, structural, diffusion, perfusion.*
Field strength	*Specify in Tesla*
Sequence & imaging parameters	*Specify the pulse sequence type (gradient echo, spin echo, etc.), imaging type (EPI, spiral, etc.), field of view, matrix size, slice thickness, orientation and TE/TR/flip angle.*
Area of acquisition	*State whether a whole brain scan was used OR define the area of acquisition, describing how the region was determined.*

Diffusion MRI ☐ Used ☐ Not used

Preprocessing

Preprocessing software	*Provide detail on software version and revision number and on specific parameters (model/functions, brain extraction, segmentation, smoothing kernel size, etc.).*
Normalization	*If data were normalized/standardized, describe the approach(es): specify linear or non-linear and define image types used for transformation OR indicate that data were not normalized and explain rationale for lack of normalization.*
Normalization template	*Describe the template used for normalization/transformation, specifying subject space or group standardized space (e.g. original Talairach, MNI305, ICBM152) OR indicate that the data were not normalized.*
Noise and artifact removal	*Describe your procedure(s) for artifact and structured noise removal, specifying motion parameters, tissue signals and physiological signals (heart rate, respiration).*

Volume censoring | *Define your software and/or method and criteria for volume censoring, and state the extent of such censoring.*

Statistical modeling & inference

Model type and settings | *Specify type (mass univariate, multivariate, RSA, predictive, etc.) and describe essential details of the model at the first and second levels (e.g. fixed, random or mixed effects; drift or auto-correlation).*

Effect(s) tested | *Define precise effect in terms of the task or stimulus conditions instead of psychological concepts and indicate whether ANOVA or factorial designs were used.*

Specify type of analysis: ☐ Whole brain ☐ ROI-based ☐ Both

Statistic type for inference | *Specify voxel-wise or cluster-wise and report all relevant parameters for cluster-wise methods.*

(See Eklund et al. 2016)

Correction | *Describe the type of correction and how it is obtained for multiple comparisons (e.g. FWE, FDR, permutation or Monte Carlo).*

Models & analysis

n/a	Involved in the study
☒	☐ Functional and/or effective connectivity
☒	☐ Graph analysis
☒	☐ Multivariate modeling or predictive analysis
