# Supplementary figures and images for: Temporal and context-dependent requirements for the transcription factor Foxp3 expression in regulatory T cells
Source: Nat Immunol. 2025 Oct 8;26(11):2059–73. doi: 10.1038/s41590-025-02295-4 (PMC12571910; doi:10.1038/s41590-025-02295-4)

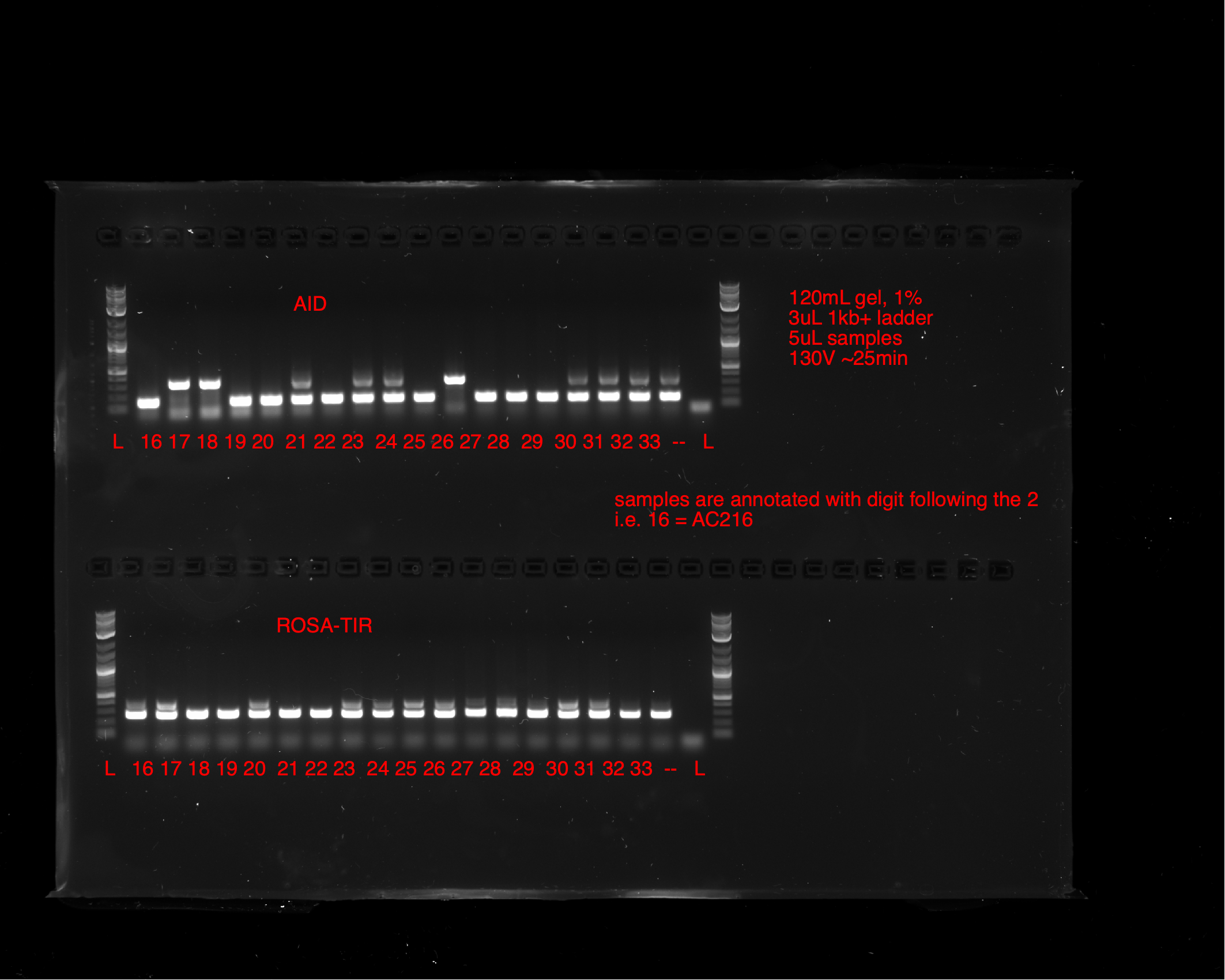

Supplement: Supplementary file 11 — Unprocessed gel image for Extended Data Fig. 1b. [file 41590_2025_2295_MOESM11_ESM.tif]

Extended Data Figure 2c

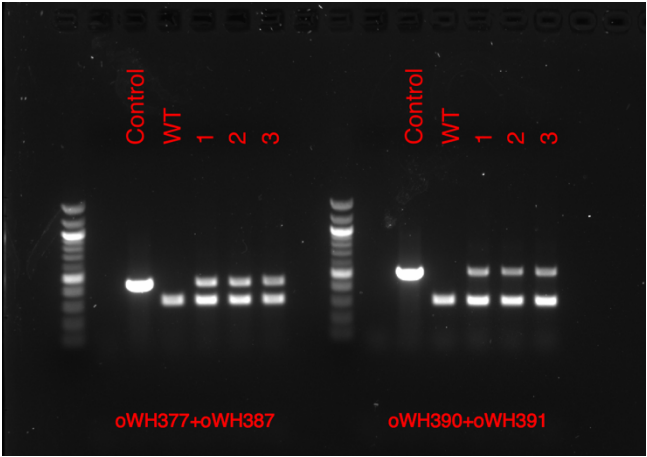

Extended Data Figure 2e

IB: Myc

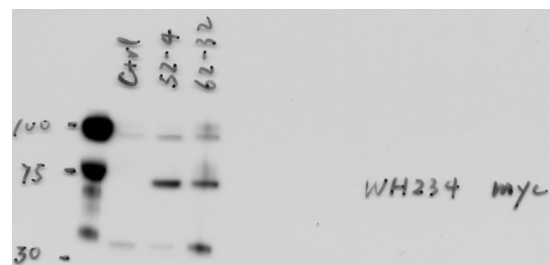

IB: GAPDH

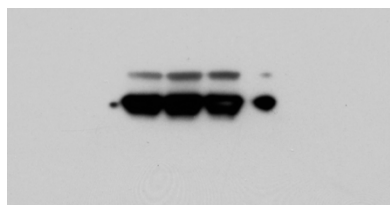

Extended Data Figure 2h

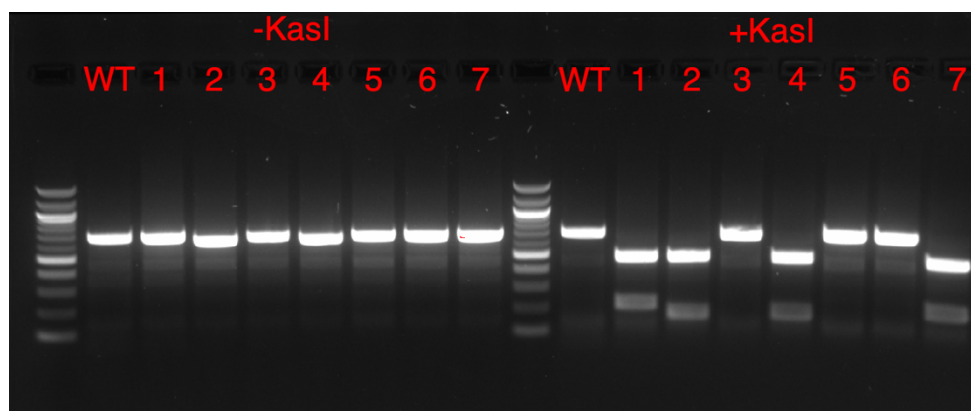

Supplement: Supplementary file 12 — Unprocessed gel image for Extended Data Fig. 2. [file 41590_2025_2295_MOESM12_ESM.pdf]
